# Therapeutic Effect of Selenium Nanoparticles, Sorafenib, and Selenium–Sorafenib Nanocomplex in the Lungs and Kidneys of Mice with TAA-Induced HCC

**DOI:** 10.3390/biom15091336

**Published:** 2025-09-18

**Authors:** Egor A. Turovsky, Sergey V. Gudkov, Elena G. Varlamova

**Affiliations:** 1 Institute of Cell Biophysics of the Russian Academy of Sciences, Federal Research Center “Pushchino Scientific Center for Biological Research of the Russian Academy of Sciences”, 142290 Pushchino, Russia; 2 Prokhorov General Physics Institute of the Russian Academy of Sciences, 38 Vavilove st., 119991 Moscow, Russia; s_makariy@rambler.ru

**Keywords:** selenium, sorafenib, hepatocellular carcinoma, thioacetamide, gene expression

## Abstract

Hepatocellular carcinoma is a primary malignant tumor of the liver, which is a serious health problem due to its aggressive nature, late diagnosis, and metastasis to other organs. We present, for the first time, the mRNA expression patterns of a wide range of genes involved in inflammation, fibrosis, endoplasmic reticulum stress, various forms of cell death, and signaling cascades in the lungs and kidneys of mice with thioacetamide-induced HCC. It is known that HCC often metastasizes to the lungs, and it is also important to understand which pathological processes occur in the kidneys, since the liver and kidneys are key target organs of toxicity. The main goal of this work was to study the pathological processes in the lungs and kidneys in HCC and the effectiveness of selenium nanocomplexes, as well as the well-known drug sorafenib, in mitigating these pathological consequences. These results present a significant contribution to the study of HCC metastasis to the lungs and kidneys and to the development of drugs that are most effective in the late stages of HCC. In addition, a hierarchy of the distribution of the selenium in the liver, kidneys, and lungs was established after the treatment of mice with HCC with selenium nanoparticles and a selenium–sorafenib nanocomplex. These data are important for developing a treatment protocol and determining optimal dosages of the drugs under study, which allows for achieving the desired therapeutic effect and neutralizing the toxic effect of selenium on healthy tissues and organs.

## 1. Introduction

Since hepatocellular carcinoma (HCC) is often diagnosed at late stages, it is extremely important to develop drugs aimed not only at leveling pathological processes in the liver but also in other organs during metastasis. Most often, metastases in HCC are observed in the lungs and lymph nodes (more than 50%), as well as in the bones (28%) [1,2]. However, since the kidneys, along with the liver, are key target organs of toxicity, it is also important to study which pathological processes occur in these organs during HCC.

To study pathological processes in the lungs and kidneys during the progression of HCC, we chose a model of thioacetamide (TAA)-induced HCC in mice. This mechanism of HCC induction is long-term and as close as possible to the progression of this disease under physiological conditions [3,4,5]. Previously, we and other authors have studied in detail the pathological processes that TAA causes in the liver during fibrosis and HCC [5,6,7,8]. It has been established that TAA causes activation of inflammatory processes due to increased expression of pro-inflammatory cytokines; can stimulate pyroptosis, oxidative stress, and endoplasmic reticulum stress (ER-stress); and activate MAPK (mitogen-activated protein kinase) signaling pathways, hypoxia, and other negative effects.

As therapeutic agents, we selected selenium (Se)-based nanoparticles (SeNPs) and a nanocomplex, the method of obtaining which we have described many times before [7,9,10,11,12,13]. It is known that the trace element selenium (Se) and compounds based on it, as well as a number of selenoproteins, have anticancer properties, which has been demonstrated in numerous studies [13,14,15,16,17,18,19,20,21]. With the development of nanotechnology, much attention in recent decades has been paid to the study of the anticancer properties of Se-based nanoparticles and nanocomplexes [22,23,24,25,26,27,28,29,30,31,32,33,34,35]. The main physical parameters influencing the pharmacokinetics and biodistribution of nanoparticles in the body are shape and size. Several studies agree that spheres are the most effective in terms of cellular uptake, mainly due to their isotropic shape, which ensures a constant distribution of acting forces and a tendency to stay in the bloodstream for a longer time [36,37,38]. In addition, spherical nanoparticles should overcome the minimum energy barrier of membrane bending compared to their non-spherical counterparts [39,40]. In addition, we have previously shown that SeNPs produced by laser ablation with an average size in the range of 90–120 nm have the most effective therapeutic properties and are non-toxic to healthy tissues and organs [10,15,24,41]. In this study, we used previously selected therapeutic doses of SeNPs, SeSo, and So, which did not have a toxic effect and did not reduce their viability [7,9,12].

In addition, a well-known first-line drug for the treatment of HCC is the multikinase inhibitor sorafenib (So), but it has a number of significant drawbacks. Some of the important drawbacks are the nonspecificity of the effect on cancer cells and toxicity with the long-term use of therapeutic doses, due to which therapy based on this drug entails a number of side effects [41,42,43,44]. Therefore, we created a nanocomplex consisting of SeNPs doped with sorafenib (SeSo) in order to reduce the range of therapeutic doses and further enhance the anticancer effect, since selenium nanoparticles themselves have well-defined anticancer properties. Previously, we tested the therapeutic effect of SeNPs, So, and SeSo on TAA-induced liver fibrosis and HCC models in mice, but our studies were limited to the liver and tumor tissue. However, at late stages of HCC, which we recorded after 8–9 months of TAA injections (200 µg/g mouse weight), the progression of pathological processes in other organs and tissues is very likely.

In this work, we conducted a study of the progression and subsequent leveling of pathological processes in the lungs and kidneys and studied the molecular mechanisms of their development, as well as the molecular mechanisms of the therapeutic effect of SeNPs, So, and SeSo. In addition, using independent approaches, we studied the accumulation of Se in the lungs and kidneys before and after treatment and compared them with similar indicators in the liver. We found that as a result of TAA injections with the powerful progression of HCC, inflammatory processes develop in the lungs and kidneys, with more pronounced pathological processes observed in the kidneys compared to the lungs. It was also shown that despite the maximum concentration of Se in the liver, especially after SeNP injections, this microelement is also able to accumulate in the lungs and kidneys and apparently have a therapeutic effect in these organs as well. Our results most likely indicate that SeNPs and SeSo, as well as to a lesser extent So, are capable of eliminating the consequences of HCC not only in the liver but also in the lungs and kidneys, which is important in the treatment of late stages of HCC.

## 2. Materials & Methods

### 2.1. Ethical Statement

The experiments were carried out on inbred mice (males) of the DBA/2J line, which were kept in a specialized vivarium of the IBC RAS. Animal housing and care complied with all recommendations of the 8th edition of the Guide for the Care and Use of Laboratory Animals by the National Research Council. Animals had unlimited access to food and water, under a 12/12 light regime, in rooms with an air exchange rate of at least 10 rpm, air temperature of 20–23 °C (daily difference of no more than 1 °C), and humidity of 30–70%. Animals were kept in cages of 10 animals, taking into account the necessary space for each mouse. A certified employee was responsible for compliance with the rules of sanitation, care, and manipulation of animals in the experiment. For the induction of HCC, a chemical method was used by intraperitoneal injections of 98% thioacetamide (#172502, «Sigma-Aldrich», St. Louis, MI, USA). Injections of TAA (200 μg/g) were carried out three times a week for 8 months. Then, for 1 month, daily injections of SeNPs, SeSo, and So were performed at a concentration of 10 μg/g of the animal. Decapitation after administration of a sedative drug to the animal was chosen as the method of euthanasia. The minimum number of animals required to obtain reliable data was used in the work (10 animals per group).

### 2.2. Animal Injection Protocols

To induce HCC, animals were injected intraperitoneally with TAA (200 μg/g mouse weight) three times a week for eight months. Following TAA injections, treatment with SeNPs, SeSo, and So was administered by intraperitoneal injections at a concentration of 10 μg/g mouse weight.

### 2.3. Isolation of Total RNA from Lungs and Kidneys, Reverse Transcription

Total RNA was isolated from tissues using the phenol-chlorophyll method with ExtractRNA (#BC032, «Evrogen», Moscow, Russia) and according to the manufacturer’s protocol. The quality of RNA extraction was checked by electrophoresis in 1% agarose gel, as well as on a spectrophotometer at a wavelength of 260 nm and by calculating the ratio of absorption values 260/280 and 260/230. To prevent contamination of the RNA samples with genomic DNA, they were treated with DNase I at 37 °C for 1 h, after which the enzyme was inactivated by adding 50 mM EDTA to the mixture and heating to 60 °C for 10 min.

To perform the reverse transcription reaction, a reagent kit (#SK021, «Evrogen», Moscow, Russia) was used; the amount of total RNA per one reverse transcription reaction was 0.5–2 μg.

### 2.4. Synthesis and Characterization of SeNPs, So, and SeSo

The protocol for the synthesis of SeNPs and the SeSo nanocomplex was developed and described by us earlier [7,9,12,13]. Briefly, a colloidal solution of selenium nanoparticles (SeNPs) was synthesized by the method of laser ablation in deionized water (resistivity of 18 MΩ·cm). A solid target placed at the bottom of a cuvette under a thin layer of water (2–3 mm thick) was irradiated with a laser beam at a wavelength of 1064 nm. A pulsed mode was used with the following parameters: pulse duration of 4–200 ns, repetition rate of 20 kHz, average power of 20 W, and pulse energy of 1 mJ. Scanning of the laser spot across the target surface was performed along a trajectory of parallel lines inscribed within a rectangle using an LScanH galvanometric scanner (Ateko-TM, Moscow, Russia).

Sorafenib (Bayer HealthCare AG, Leverkusen, Germany) was immobilized on the surface of the nanoparticles as follows: A solution of the drug was prepared in citrate buffer (pH 4.1), as its solubility is pH-dependent and decreases in an alkaline environment. Selenium nanoparticles were added to the sorafenib solution, and the mixture was incubated for 30 min. Following incubation, centrifugation was performed to separate the nanoparticles from the solution. The amount of the sorbed drug was determined spectrophotometrically by measuring the change in the optical absorption of the supernatant before and after nanoparticle precipitation. The experiment was repeated in triplicate.

After the adsorption of sorafenib, the hydrodynamic radius of the nanoparticles increased, and the zeta potential rose to a value of −23 mV. Direct microscopic examination of the modified nanoparticles was not performed due to the low contrast of the organic sorafenib layer.

**Figure 1:**
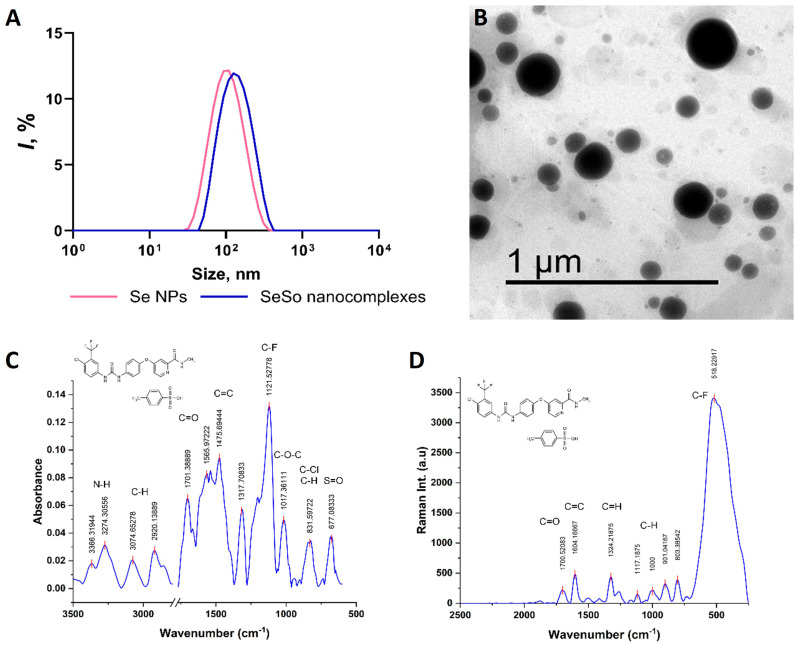
Characteristics of SeNPs, SeSo, and sorafenib: TEM image of Se NPs (**A**); hydrodynamic size distributions of Se NPs and SeSo nanocomplexes (**B**); FTIR (**C**) and Raman (**D**) spectra of sorafenib tosylate used for the synthesis of SeSo nanocomplexes. The chemical structure of sorafenib is shown in the upper left corner of the spectra.

The SeNP complexes used to obtain the SeSo nano-complex exhibited a monomodal size distribution. The average hydrodynamic diameter of the SeNPs was approximately 110 nm, while for the SeSo nanocomplexes, an increase in the average hydrodynamic diameter to approximately 125 nm was observed (Figure 1A, Supplementary Figures S1 and S2). The transmission electron microscopy (TEM) results confirmed that the obtained SeNPs were spherical in shape (Figure 1B). Fourier-transform infrared (FTIR) spectroscopy and Raman spectroscopy were used to obtain the spectra of the pure sorafenib (So) used for the synthesis of the SeSo nanocomplexes (Figure 1C,D).

### 2.5. Real-Time PCR

A reagent kit containing SYBRGreen (#PK147, «Evrogen», Moscow, Russia) was used to perform real-time PCR reactions. Amplification was performed according to the following temperature regime: 95 °C-1 min; 95 °C-10 s, 60 °C-10 s, and 72 °C-15 s (35–40 cycles). Fluorescence was removed during the elongation stage; the melting curve increased from 60 to 95 °C, with a step of 0.3 °C. The relative gene expression level (RGE—the expression level of the studied gene relative to the expression of the reference gene) in each cell line was determined by the formula RGE = 2^−ΔCt^, where ΔCt is the difference between the threshold cycle values for the reference and target genes. The change in the mRNA expression level of the studied proteins before and after treatment was determined by the following formula: RGE = 2^−ΔΔCt^, where ΔΔCt is the difference in ΔCt values for each gene before and after cell treatment. Each cycle of the experiment was repeated three or more times. GAPDH (glyceraldehyde-3-phosphate dehydrogenase) was used as a reference gene for the real-time PCR. The sequences of the primers used in the work are given in Table 1.

### 2.6. Western Blotting

To obtain protein lysates, tissue weighing 100–200 mg was ground under liquid nitrogen and then lysed in a special buffer (#IS007, «Proteins-Antibodies», Moscow, Russia). Then, tissue lysis samples were centrifuged at 5–7000 rpm to remove the undissolved homogenate, after which the lysis samples were concentrated using protein concentrators (#88517, «Thermo FS», Waltham, MA, USA). PAGE was performed in 10 or 12.5% polyacrylamide resolving gel, adding 100 μg of total protein per well. Protein bands were transferred from the gel to PVDF membranes (#LC2005, «Thermo FS», Waltham, MA, USA), after which the membrane was packed in 5% BSA for 5–6 h at 4 °C. Then, the membranes were incubated with primary antibodies for 16 h at 4 °C in a dilution of 1:500. The following primary antibodies were used in the work: anti-GAPDH (#14–9523–82, «Thermo FS», Waltham, MA, USA), anti- anti-CASP-3 (Abcam, Cambridge, UK, #ab184787), anti-IL-1β (Abcam, Cambridge, UK, #ab205924), anti-IL-6 (Abcam, Cambridge, UK, #ab290735), anti-IL-33 (Abcam, Cambridge, UK, #ab187060), anti-active CASP-1 (Invitrogen, Carlsbad, CA, USA, #PA5–77886), anti-XBP1 (Invitrogen, #PA5–27650), anti-ATF-4 (Invitrogen, #PA5–19521), anti-ATF-6 (Invitrogen, #PA5–20216), and anti-α-SMA (Abcam, Cambridge, UK, #ab7817). After thoroughly washing the membranes in 1x PBST, they were incubated with secondary antibodies for 2 h at room temperature. The following secondary antibodies were used in the work in a dilution of 1:5000: rabbit anti-mouse Ab (Abcam, Cambridge, UK, #ab6728) and mouse anti-rabbit Ab (Abcam, Cambridge, UK, #ab99697). Then, they were washed thoroughly and immunoreactive bands were visualized by determining the peroxidase activity using DAB staining (0.05% DAB in 1x PBS + 10 μL 30% hydrogen peroxide). Immunoreactive bands were visualized by determining the peroxidase activity using DAB staining (0.05% DAB in TBS + 10 μL 30% hydrogen peroxide).

### 2.7. Quantitative Assessment of Se Contents in the Lungs, Kidneys, and Liver by the Colorimetric Method

This procedure was carried out using the Selenium Assay Kit (#abx298910 «Abbexa Ltd.», Cambridge, UK) according to the manufacturer’s protocol. Tissue pieces weighing approximately 100 mg were homogenized in 250 μL of assay buffer 1 and thoroughly mixed on a vortex. Then, 250 μL of assay buffer 2 were added and mixed again. After, the tubes with samples were centrifuged at 13,000 rpm for 10 min at 4 °C. The supernatant was transferred to new tubes and then loaded onto a 96-well plate with the addition of 50 μL of reaction buffer 1, 50 μL of reaction buffer 2, and 90 μL of Due reagent solution. After loading, the plate was incubated on a shaker in a thermostat at a temperature of 90 °C. Registration was performed using an iMark microplate absorbance reader («Bio-Rad», Hercules, CA, USA) at a wavelength of 520 nm. Measurements were carried out in duplicate for each sample. The intensity of the color is proportional to the concentration of selenium, which can then be calculated.

### 2.8. Quantitative Assessment of Se Contents in Lungs, Kidneys, and Liver by Hydride Generation Atomic Absorption Spectrometry (HG-AAS)

Samples of lung, kidney, and liver tissues weighing 200–500 mg were placed in fluoroplastic vessels and subjected to acid mineralization at an elevated pressure using microwave heating in sealed vessels. The mineralization program included a two-stage increase in temperature to 195 °C and cooling to room temperature. After 60 min, the mineralizate was transferred to a measuring tube and diluted with deionized water, recording the total volume. To convert all forms of selenium to a form suitable for hydrogenation—Se (IV)—5 cm^3^ of concentrated HCl and 5 cm^3^ of the solution obtained after microwave mineralization were added to a 10 cm^3^ test tube. The tubes were heated in a water bath for 20 min at a temperature of no more than 90 °C. After cooling the tubes to room temperature, the volume was brought to 10 cm^3^ with 6 M HCl. Then, using a standard solution of selenium (IV) GSO 7340–96, the atomic absorption spectrometer Varian AA240FS («EquipNet», New South Wales, Australia) with a VGA-77 hydride attachment was calibrated, and subsequent measurements of the mass concentration of selenium in the mineralizates were made at a wavelength of 196 nm. The mass fraction of selenium in the sample (mg/kg) was calculated using the following formula: X = C*F*V/1000*m, where C is the mass concentration of selenium in the analyzed solution, μg/dm^3^; F is the sample dilution factor; V is the volume of the sample solution after mineralization, cm^3^; 1000 is the conversion factor from μg to mg; and m is the mass of the sample, g. The arithmetic means of three measurements with an expanded uncertainty of no more than 35% is taken as the measurement result.

### 2.9. Statistical Data Processing

Microsoft Excel and GraphPadPrism 5 were used to analyze the data, create graphs, and process statistics. Protein was assessed in different samples using the Lowry method. The protein concentration was calculated using a standard curve constructed using 1 mg/mL BSA solution. Values are presented in the paper as the mean ± standard deviation of at least three independent experiments. Differences were considered significant at *p* < 0.05. Protein expression was quantified using ImageJ Version 1.54p. The resulting time series were processed in ImageJ with the Time Series Analyzer plugin. Origin 8.5 («Microcal Software Inc.», Northampton, MA, USA) and Prism 5 («GraphPad Software», La Jolla, CA, USA) were used to construct graphs and perform statistical processing. The reliability of the differences between experimental groups was determined using variance analysis (one-way ANOVA with a post hoc Tukey test or post hoc Student–Newman–Keuls test) or the Student’s *t*-test, and for within groups the Student’s *t*-test was used. Differences were considered reliable according to the following: *** at *p* < 0.001, ** at *p* < 0.01, * at *p* < 0.05, and n/s—differences are not reliable.

## 3. Results and Discussion

### 3.1. SeNP Injections Promote the Most Effective Accumulation of Se in the Liver, Lungs, and Kidneys Compared to SeSo

To understand how selenium-based nanoparticles selectively exert their therapeutic effect on tissues in which pathological processes progress, it was necessary to evaluate the concentration of this microelement in the liver, as well as in the lungs and kidneys, after SeNPs and SeSo injections and compare these parameters measured after TAA and So injections. To determine the Se concentrations in the liver, lungs, and kidneys before and after TAA, So, SeNPs, and SeSo injections, we carried out two independent approaches.

**Figure 2:**
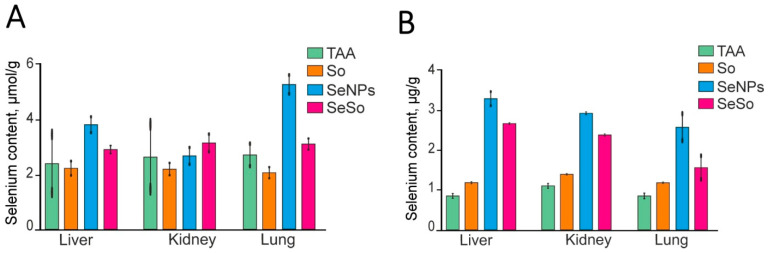
Quantitative assessment of Se contents in the liver, lungs, and kidneys of mice with TAA-induced HCC and after injections of So, SeNPs, and SeSo using the colorimetric method (**A**) and hydride generation atomic absorption spectrometry (HG-AAS) method (**B**).

In the first case, the Se concentration in the studied organs was determined using the colorimetric method, a detailed description of which is given above. As a result of this analysis, we found that the Se concentration in tissue samples after TAA injections significantly increased compared to the control samples (healthy mice) only in the lungs (Figure 2A). Subsequent injections of SeNPs contributed to a significant increases in the Se concentrations in the liver and especially in the lungs. The increases in the Se concentrations in all three organs were significantly less intense after SeSo injections, while So, as expected, did not significantly affect these indicators in any of the studied organs. The increase in the Se concentration in the lungs even after TAA injections (i.e., during the progression of the late stages of HCC) can be explained by the fact that Se can affect the proliferation of tumor cells by changing the state of glutathione in them, which is important for tumor development [15]. One of the reasons for Se toxicity in cancer cells compared to healthy somatic cells is their more reducing environment, which stimulates the formation of Se-S adducts from Se compounds. Such Se-S adducts can mimic cystine and mixed disulfides, causing enhanced receptor-mediated Se uptake by cancer cells [45,46,47,48,49].

In the colorimetric method, registration was performed at a wavelength of 520 nm. Measurements were carried out in duplicate for each sample. The intensity of the color is proportional to the concentration of Se, which can then be calculated. In HG-AAS, the mass fraction of Se in the sample (μg/g) was calculated using the following formula: X = C*F*V/1000*m, where C is the mass concentration of Se in the analyzed solution, μg/dm^3^; F is the sample dilution factor; V is the volume of the sample solution after mineralization, cm^3^; 1000 is the conversion factor from μg to mg; and m is the mass of the sample, g. The arithmetic means of three measurements with an expanded uncertainty of no more than 35% is taken as the measurement result.

The second method we used to determine the Se content in various organs was hydride generation atomic absorption spectrometry (HG-AAS). According to the results, it can be said that SeNPs most effectively caused increases in the Se concentrations in the liver, lungs, and kidneys compared to SeSo (Figure 2B). Here, a slight increase in the Se concentration in all organs after TAA injections was also noted. This may contribute to enhanced biosynthesis of selenium-containing proteins. We previously showed that in HCC induced by TAA injections, the mRNA expression patterns of selenium-containing thioredoxin reductases TXNRD1 and TXNRD2, glutathione peroxidases GPX1 and GPX2, and deiodinase DIO3, as well as selenoproteins SELENOP and SELENON, were enhanced in the liver [50]. In recent decades, it has been repeatedly proven that mammalian selenoproteins play an important role in carcinogenesis, and their expression changes significantly in cancers of various etiologies [15,16,17,18,19,20,21,50,51].

### 3.2. Injections of SeNPs, So, and SeSo Effectively Reduce the Inflammatory Status and Fibrotic Changes in the Liver and Lungs in Mice with TAA-Induced HCC

**Figure 3:**
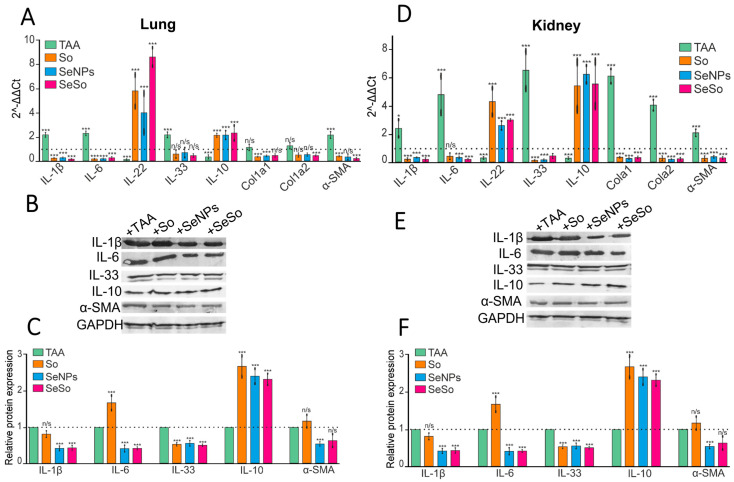
mRNA expression patterns of inflammatory markers and their relative amounts in the lungs and kidneys after injection of TAA, So, SeNPs, and SeSo. (**A**,**D**)—real-time PCR results. The mean values ± standard errors (SEs) were determined by analyzing data from at least three independent experiments and are indicated by error bars; n/s—data are not significant (*p* > 0.05), * *p* < 0.05 and *** *p* < 0.001. (**B**,**E**)—immunoblotting results. (**C**,**F**)—quantitative assessment of inflammatory markers in kidney and lung samples obtained using ImageJ, presented as the mean ± standard deviation of three independent experiments. GAPDH was used as a control for normalization. The original Western blots are shown in Supplementary Figure S3.

It is well known that the progression of HCC is accompanied by an increase in the inflammatory status in hepatocytes. In this work, we tested how the inflammatory status in the lungs and kidneys changes during the development of TAA-induced HCC. According to the results of the real-time PCR and Western blot analysis, the mRNA expression of some studied pro-inflammatory markers increased after TAA injections both in the kidneys and lungs by two or more times (Figure 3A–F).

Thus, increases in the mRNA expression of pro-inflammatory cytokines IL-1β, IL-6, and IL-33 were observed, while the levels of IL-22 and IL-10 mRNA expression, on the contrary, decreased. IL-22 is considered to be a protective cytokine for the liver, promoting tissue regeneration, enhancing barrier function, reducing chronic inflammation, and preventing cancer [52]. The anti-inflammatory cytokine IL-10 promotes antitumor immunity, thereby inhibiting tumor growth [53,54]. Therefore, the trend toward a decrease in their mRNA expression levels and quantities in lung and kidney cells after TAA injections may have negative consequences, contributing to the progression of inflammatory reactions in these organs. Subsequent injections of SeNPs, So, and SeSo contributed to decreases in IL-1β and IL-6 mRNA expression in the lungs and kidneys (Figure 3A,D). However, according to the results of Western blotting, SeNPs and SeSo were the most effective at reducing the levels of the relative contents of these proteins (Figure 3B,C,E,F). In addition, in the kidneys, all three “drugs” reduced the amount of IL-33 at both the transcriptional and translational levels. At the same time, none of the studied “drugs” significantly affected the levels of IL-33 mRNA and protein in the lungs. In both studied organs, injections of the three drugs contributed to increases in the levels of mRNA and the amounts of IL-22 and IL-10 cytokines.

In addition, we examined the expression of a number of markers that are typically upregulated in fibrotic changes. Since it is well known that tissue fibrosis, which is characterized by excessive connective tissue formation, may be associated with an increased risk of cancer in some cases, COL1A1 and COL1A2 are known to encode the alpha1(I) and alpha2(I) chains of type I collagen, respectively. As fibrosis progresses, COL1A1 and COL1A2 are involved in the deposition of excess collagen in tissues, leading to stiffness and dysfunction [55]. Increased expression of alpha-smooth muscle actin (α-SMA) mRNA is also often indicative of increased collagen synthesis and fibrosis [56]. According to our real-time PCR data, we observed increases in the expression levels of α-SMA mRNA after TAA injections in the lungs and kidneys, while sharp increases in COL1A1 and COL1A2 were observed only in the kidneys. These data may indicate the activation of fibrotic processes in these organs. Subsequent injections of SeNPs, So, and SeSo contributed to decreases in these indicators compared to control values (healthy animals).

Thus, long-term TAA injections lead not only to the progression of HCC but also to slight increases in the inflammatory statuses in the lungs and especially in the kidneys. Subsequent injections of SeNPs, So, and SeSo contributed to decreases in the levels of pro-inflammatory cytokines in the lungs and kidneys compared to control values. In addition, all three drugs contributed to growths in the anti-inflammatory cytokines IL-22 and IL-10, as well as reduced the mRNA expression levels of α-SMA, COL1A1, and COL1A2 in the studied organs. All of this indicates that the negative consequences of long-term TAA injections, which led to the progression of HCC, were most pronounced in the kidneys compared to the lungs. However, subsequent treatment with the studied drugs, especially SeNPs and SeSo, significantly neutralized inflammation and fibrosis in these organs (Figure 3).

### 3.3. Injections of SeNPs, So, and SeSo Effectively Reduce Apoptotic Cell Death in the Lungs and Kidneys of Mice with TAA-Induced HCC

We have previously shown that TAA can induce oxidative stress in the liver, as well as endoplasmic reticulum stress, which is initially adaptive in nature but becomes prolonged as inflammation and fibrosis progress, leading to increased ER load and apoptotic death of hepatocytes [7]. In this study, we also tested how the expression of mRNA for key markers of three adaptive response signaling pathways changes during ER-stress, as well as monitored for the presence of signs of apoptotic cell death in the lungs and kidneys.

**Figure 4:**
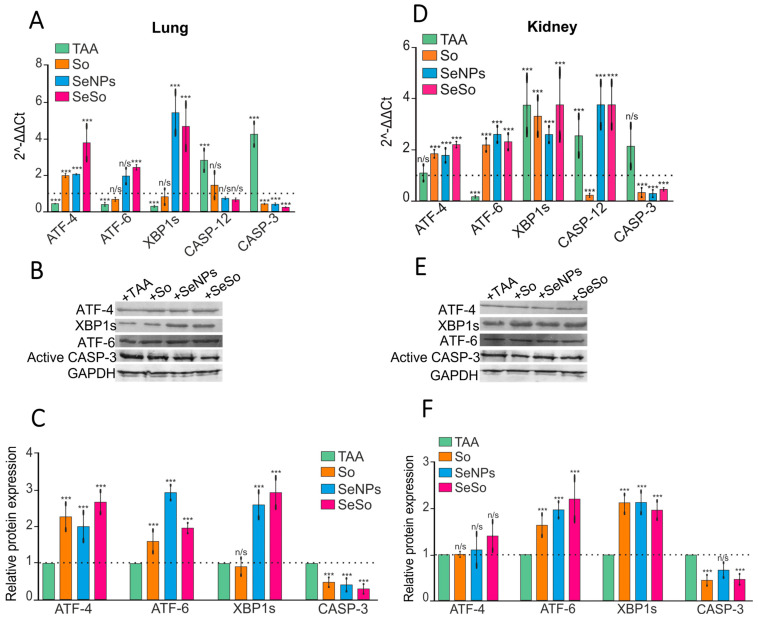
mRNA expression patterns of ER-stress and apoptosis markers and their relative amounts in the lungs and kidneys after injections of TAA, So, SeNPs, and SeSo. (**A**,**D**)— real-time PCR results. The mean values ± standard errors (SEs) were determined by analyzing data from at least three independent experiments and are indicated by error bars; n/s—data are not significant (*p* > 0.05) and *** *p* < 0.001. (**B**,**E**)—immunoblotting results. (**C**,**F**)— quantitative assessment of ER-stress and apoptosis markers in kidney and lung samples obtained using ImageJ, presented as the mean ± standard deviation of three independent experiments. GAPDH was used as a control for normalization. The original Western blots are shown in Supplementary Figure S3.

The results of the real-time PCR and Western blot analysis shown in Figure 4 indicate changes in the expression patterns of markers of the following three misfolding response (UPR) signaling pathways: activating transcription factors 4 and 6 (ATF-4, ATF-6) and X-box-binding protein 1 (XBP1s) (Figure 4). In the kidneys, a sharp decrease in the transcription factor ATF-6 was observed, but an increase in XBP1s, as well as two caspases CASP-12 and CASP-3, was recorded. In the lungs, after TAA injections, decreases in the expression patterns of all three transcription factors—ATF-4, ATF-6, and XBP1s—were observed, but the expression of mRNA and the relative contents of the studied caspases tended to increase. This may indicate that under the influence of TAA in the kidneys and lungs, an increase in prolonged ER-stress was observed, which ultimately led to the activation of effector caspase-3 and, consequently, an increase in the likelihood of apoptotic cell death. The increase in CASP-12 mRNA expression under ER-stress conditions is apparently carried out through the Ca^2+^-signaling pathway, which also leads to the activation of caspase-3 [55,56]. Subsequent injections of sorafenib resulted in a decrease in the expression of both caspases in the kidneys, while we observed a trend toward increases in the expression of two ER-stress markers, ATF-6 and XBP1s. After injections of SeNPs and SeSo, a decrease in caspase-3 was also observed, but increases in CASP-12 and other UPR transcription factors were observed. Thus, after injections of SeNPs, an increase in the expression of the MRNA of the transcription factor ATF-6 and the expression level of the spliced form of mRNA XBP1s of the transcription factor were observed in the kidneys, while after injections of SeSo, increases in the expression of the MRNA of all three transcription factors were noted in the kidneys. In the lungs, after injections of sorafenib, a decrease in the expression of the MRNA of caspase-3 and a slight increase in the expression of the MRNA of the transcription factor ATF-4 were observed, while after injections of SeNPs and SeSo, against the background of a decrease in the expression of the MRNA of caspase-3, we observed increases in the expression of the MRNA of all three transcription factors. The results obtained by the real-time PCR method were confirmed by the analysis of the quantitative assessment of proteins encoded by the studied genes (Figure 4B,C,E,F). It can be assumed that the increase in the expression of the studied transcription factors activated during ER-stress against the background of a decrease in CASP-3 is adaptive. In other words, the load on the ER after injections of So, SeNPs, and SeSo is significantly reduced by decreasing protein misfolding as a result of the activation of these transcription factors, which has been repeatedly proven previously [7,57,58,59,60,61,62,63,64,65,66,67,68,69,70].

### 3.4. Analysis of the Screening of mRNA Expression Patterns of Markers of Various Signaling Pathways in the Kidneys and Lungs of Mice with HCC Induced by TAA and After Injections of So, SeNPs, and SeSo

**Figure 5:**
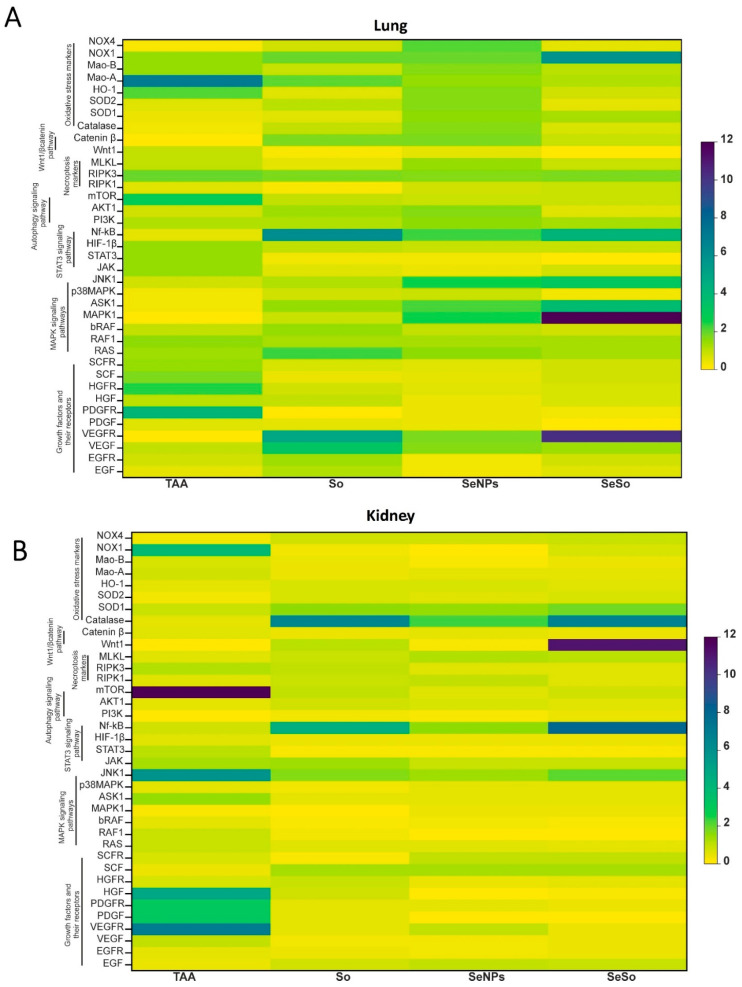
Heat maps of the screening of mRNA expression patterns of markers of various signaling pathways in the lungs (**A**) and kidneys (**B**) of mice with HCC induced by TAA and after injections of So, SeNPs, and SeSo. Mean values ± standard errors (SE) were determined by analyzing data from at least three independent experiments. GAPDH was used as a control for normalization.

We conducted a large-scale screening of expression patterns of markers of various signaling cascades, growth factors and their receptors, antioxidants both after TAA injections and after injections of So, SeNPs, and SeSo. Based on the obtained results, we compiled heat maps of expression patterns, which are presented in Figure 5A,B. The analysis of the expression patterns presented in the heat maps suggests that in HCC induced by prolonged TAA injections, the mRNA expression patterns of the studied genes are different in the lungs and kidneys. In the kidneys, we can observe increases in the expression of the MRNA of growth factor genes and their receptors, which may indicate the progression of pathological processes in them. It is known that disruption of the regulation of signaling pathways involving tyrosine kinase receptors, which are activated upon binding to specific growth factors or hormones, is associated with the progression of HCC [71,72,73,74,75,76]. In the kidneys, increases in the levels of mRNA expression of VEGFR (vascular endothelial growth factor receptor), PDGF and PDGFR (platelet-derived growth factor receptor), and HGF (hepatocyte growth factor) were observed (Figure 5B). In the lungs, it can also be observed that the TAA injections caused an increase in the expression of the mRNA of the platelet-derived growth factor receptor (PDGFR) (Figure 5A). Since growth factors and their receptors play an important role in the development of cancer [77,78,79,80,81,82,83,84,85], participating in the processes of cell proliferation and differentiation and being the cause or consequence of tumor transformation, a decrease in their expression by subsequent injections of the studied “drugs”, especially SeNPs and SeSo, is another important therapeutic effect.

## 4. Conclusions

Thus, SeNPs not doped with So contributed to better Se accumulation in all of the organs studied, especially in the liver. Apparently, an increase in the size of nanoparticles due to doping them with So somewhat reduces their penetrating ability into cells. We have shown that in the lungs and kidneys after 8 months of TAA injections, there was a trend toward the development of pathological processes, such as an increase in the inflammatory status, progression of fibrogenesis, risk of apoptotic cell death, and increase in the expression of the MRNA of the growth factors and receptors for them. The subsequent increase in the Se concentration in these organs, especially after SeNP injections, is not accidental but specific, since in this study we established their significant therapeutic effect. Despite the fact that the Se concentration after injections of the SeSo nanocomplex contributed to a slightly smaller increase in the Se concentration in the tissues studied, their therapeutic effect was comparable to the effect of SeNPs. Most likely, the range of Se concentrations observed after injections of both Se-containing agents is sufficient to provide a therapeutic effect on the lungs and kidneys. In addition, given that the SeSo nanocomplex is slightly larger in size than SeNPs, they may be able to provide a prolonged effect. All three therapeutic agents under study (So, SeNPs, and SeSo) showed a significant therapeutic effect, leveling the progression of pathological processes in the kidneys and lungs in HCC caused by TAA injections. Our further studies will be aimed at studying the pharmacokinetics, bioavailability, and biodistribution of 100 nm spherical SeNPs not doped with sorafenib in the treatment of HCC, not only in the lungs and kidneys but also in other organs and tissues. Histological and immunohistological studies of organ tissues before and after TAA injections and subsequent SeNP injections will be carried out. We hope that these studies will expand our understanding of HCC metastasis in the body, the accumulation of SeNPs in various organs during treatment, the degree of their toxicity, and the effectiveness of the fight against metastases.

## Figures and Tables

**Table 1:** Oligonucleotide sequences.

Gene	Forward Primer 5′-> 3′	Reverse Primer 5′-> 3′
*α-SMA*	AGGAAGGATCTCTATGCTAACAAC	ACTTAGAAGCATTTGCGGTGG
*COL1a1*	CATCACCTATCACTGCAAGAAC	AGGTCTTGGTGGTTTTGTTATTC
*COL1a2*	TCTCAGAACATCACCTACCAC	CACGGAATTCTTGGTCAGCAC
*IL-1β*	CGTGCTGTCGGACCCATATG	GCTCTTGACTTCTATCTTGTTG
*IL-6*	TCCAGAGATACAAAGAAATGATG	TTGGAAATTGGGGTAGGAAGG
*IL-22*	GCTCCCCCAGTCAGACAGG	TAGAAGGCAGGAAGGAGCAG
*IL-33*	TTTTGGAGAATGGATGTTATGTG	TTTGTGAAGGACGAAGAAGGC
*IL-10*	AGCATGGCCCAGAAATCAAGG	AGACTCAATACACACTGCAGG
*CASP-1*	AGAGAAATGAAGTTGCTGCTGG	ATCACCTTGGGCTTGTCTTTC
*BAK*	CACAGCCGGGAATGCCTAC	TCAGGATGGGGTCTCTACGC
*BAX*	TTCAACTGGGGCCGCGTGG	TTCCAGATGGTGAGCGAGGC
*PUMA*	TGAAGATCTGCGCCGGGAG	GAGAGGGACATGACGCGTG
*BIM*	AATGGCCGGCTATGGATGATG	GCCAATTGGGTTCACTGTCTG
*CHOP*	CAGCTGGGAGCTGGAAGCCTG	GACCACTCTGTTTCCGTTTCC
*CASP-3*	CTCTTCATCATTCAGGCCTGC	GACCCGTCCTTTGAATTTCTC
*BCL-2*	AAGTCAACACAAACCCCAAGTCCTC	GCAGATCTTCAGGTTCCTCCTGAGA
*ATF4*	TCGGGTTTGGGGGCTGAAG	AAACAGAGCATCGAAGTCAAAC
*ATF6*	AGGAGGGGAGATACGTTTTAC	CGAGGAGCTTTTGATGTGGAG
*XBPs1*	AGTCCGCAGCACAGCAGGT	AGAGAAAGGGAGGCTGGTAAG
*CASP-12*	TGTTGGTGTTATCATTTGGAGG	TTTTCTTTTCTTCTCAGCTACAG
*PI3K*	TGGCTGGGGAATGAAAATACC	AGGGAGCTGTACAGGTTGTAG
*AKT1*	ATGTGTATGAGAAGAAGCTGAG	GCGGGGCTTCTGGACTCGG
*mTOR*	TGGCCAGTCAGTCGAAATTTTG	AGTTACCAGAAAGGGCACCAG
*RIPK1*	TTGGAACTACAGGTACAGGAG	GGGTTCAGGTGTTCATCAGTC
*RIPK3*	TTCGATGGCCCAACCTCCC	TGCCCGAAGGTGCCAAGCC
*MLKL*	AAGATCCCATTTGAAGGCTGTG	GGCTCATGGGCACGACACTC
*CAT*	TGCGGACATTCTACACAAAGG	CGGAGTTACAGGTTAGCTTTTC
*SOD1*	TGGGGACAATACACAAGGCTG	ATCTTGTTTCTCATGGACCACC
*SOD2*	GGAGAGTTGCTGGAGGCTATC	GAAGGTAGTAAGCGTGCTCC
*HO-1*	AGGGTGACAGAAGAGGCTAAG	AATTCCCACTGCCACTGTTGC
*MAO-A*	TGAATGCTCTAGGAAAAGTTGC	AATTCATCCTCACTTTCCTTTAC
*MAO-B*	GGCTGCTACACAACCTACTTC	GGTAATGGGTCGTGCAGGGA
*NOX1*	ACAAGAGATGGAGGAATTAGG	TTCCTAGGATCCAGACTCGAG
*NOX4*	TACCTCAGTCAAACAGATGGG	TGTCCCATATGAGTTGTTCCG
*JAK*	CGAGAAGAGTAAAAGTCCACC	GAGCTTTGTTCTGGTTCTGGA
*STAT3*	CCCCGTACCTGAAGACCAAG	ATGGGGTTCGGCTGCTTAGG
*HIF1α*	GGCGACTGTGCACCTACTATG	TGATCCAAAGCTCTGAGTAATTC
*NFkB*	TTAAAGAAACACTCAACAGCCAG	TTCAGCACTCGCACGGACAC
*RAS*	AACAAGTGTGACCTGGCTGC	TCCGGCACCTCCATGTCCTG
*RAF1*	CCCACATCAACAACCGAGAC	CGATGCAGGGAAGGCTCAG
*bRAF*	CTGCTGGCCCGCTCATTGC	TAAGAAATAAAGAGTAGATGCTGC
*MAPK1*	GGAGCAGTATTATGACCCAAG	GCTGAGACGGGCTGAAGAC
*ASK1*	CCTAACAAGAACAGACACCCC	GGGCAGGGGATTGGAGTGG
*p38MAPK*	CTGTCGACCTACTGGAGAAG	TAGACAGAACAGAAACCAGGTG
*JNK1*	AAGCCCCACCACCAAAGATC	TCTGTATCCGAGGCCAAAGTC
*WNT*	GTTCATCTTCGCAATCACCTC	GGCATTTGCACTCTTGGCGC
*CATENINβ*	GGGGTCCTCTGTGAACTTGC	GCACCAATGTCCAGTCCAAG
*EGF*	CCTTGGTTTGTGGTCCTAGAG	CTGGGGTCCTCTGTCACTTG
*EGFR*	ATGGAGGATGTAGTTGATGCTG	ACATATTCAGGTACAGGGAGG
*VEGF*	CCCGGTTTAAATCCTGGAGC	CTTTCCGGTGAGAGGTCTGG
*VEGFR*	TGCAGATCCACATTTTCATTCC	CTTGCTTTTACTCGCTATTCTC
*PDGF*	ATTGAGATTGTGCGAAAGAAGC	GGGGGCAATACAGCAAATACC
*PDGFR*	GCCGTGCAGCCCAATGAGAG	GGCTCTGCTTGCTGTGGCTC
*HGF*	TACAATTGATTTACCTAGTTATGG	ACCATTCTCATTTTGTGTTGTTC
*HGFR*	CTCTTATCCCGACGTGAACAC	ATCATGTGTTCCCCTCGCCA
*SCF*	CCAAAATGCTGCGGAGTAATG	GTGTTATTTTTGTCCAAGCTGTG
*SCFR*	GGAAGCAGCCCCTACCCAG	CACGGGGTTCTCTGGGTTG
*GAPDH*	GTAAAGACCTCTATGCCAACAC	GGTGCACGATGGAGGGGC

## Data Availability

The data presented in this study are available upon request from the corresponding author.

## Supplementary Materials

The following supporting information can be downloaded at https://www.mdpi.com/article/10.3390/biom15091336/s1. Figure S1: Characteristics of SeNPs and functionalized selenium nanoparticles with sorafenib (SeSo): distribution of hydrodynamic diameter of nanoparticles (a), TEM micrograph of SeNPs (b), distribution of ζ-potential (c), UV-Vis absorption spectra for So, SeNPs and SeSo (d). Figure S2: Fluorescence maps of SeNPs (a), SeSo nanocomplexes (b) and sorafenib (So) (c). Figure S3: The original Western blots.
