# Supplementary material for: Therapeutic Effect of Selenium Nanoparticles, Sorafenib, and Selenium–Sorafenib Nanocomplex in the Lungs and Kidneys of Mice with TAA-Induced HCC
Source: Biomolecules. 2025 Sep 18;15(9):1336. doi: 10.3390/biom15091336 (PMC12467918; doi:10.3390/biom15091336)
Supplement: Supplementary file 1 [file biomolecules-15-01336-s001.zip › biomolecules-3779948-supplementary.pdf]

The average size of Se NPs synthesized by laser ablation was about 90–100 nm, after functionalization with sorafenib their size increased by ~10–15 nm (Figure 1a). According to TEM data, the synthesized Se NPs had a spherical morphology (Figure 1b). Functionalization of Se NPs with sorafenib led to a change in the distribution of the ζ-potential of the nanoparticles: from -5 mV for Se NPs to -25 mV for Se-So NPs (Figure 1c). Analysis of optical absorption in the UV and visible regions confirmed the formation of the sorafenib-Se NPs complex. The absorption spectrum of pure Se NPs demonstrated a uniform profile without pronounced peaks in the short-wavelength region, with a maximum in the range of 610–620 nm. At the same time, the spectrum of Se-So nanocomplexes was characterized by intense absorption in the range of 240–270 nm, which corresponds to the spectral features of sorafenib (Figure1d).

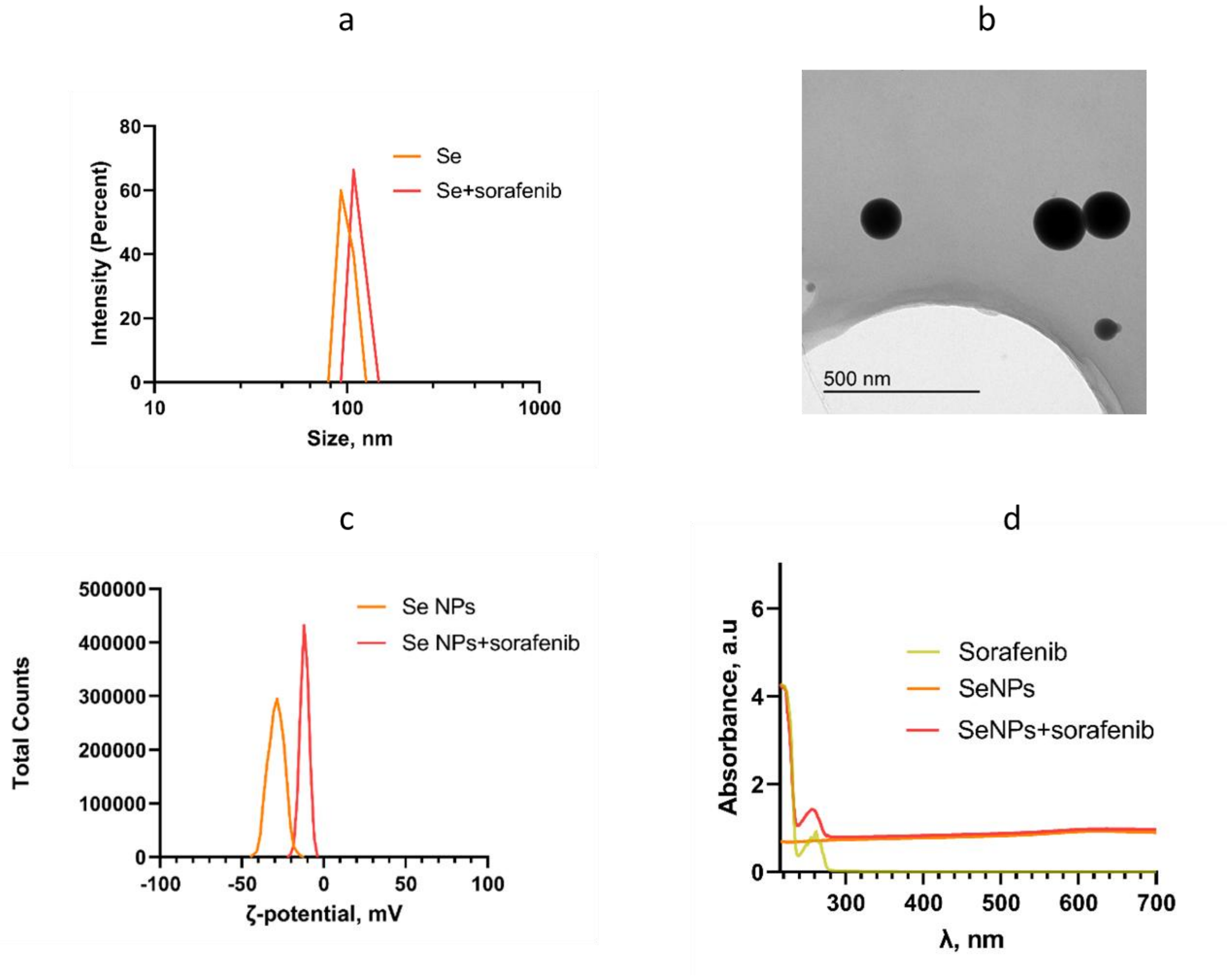

Supplementary Figure S1. Characteristics of SeNPs and functionalized selenium nanoparticles with sorafenib (SeSo): distribution of hydrodynamic diameter of nanoparticles (a), TEM micrograph of SeNPs (b), distribution of ζ-potential (c), UV-

Vis absorption spectra for So, SeNPs and SeSo (d) Analysis of the fluorescent properties of Se NPs and Se-So nanocomplexes demonstrated that the colloidal solution of selenium nanoparticles exhibited virtually no fluorescence (Figure 2a). At the same time, in the case of Se-So nanocomplexes, pronounced fluorescence was observed upon excitation in the range of 240–275 nm, with an emission maximum at about 290 nm (Figure 2b). This effect is due to the presence of sorafenib

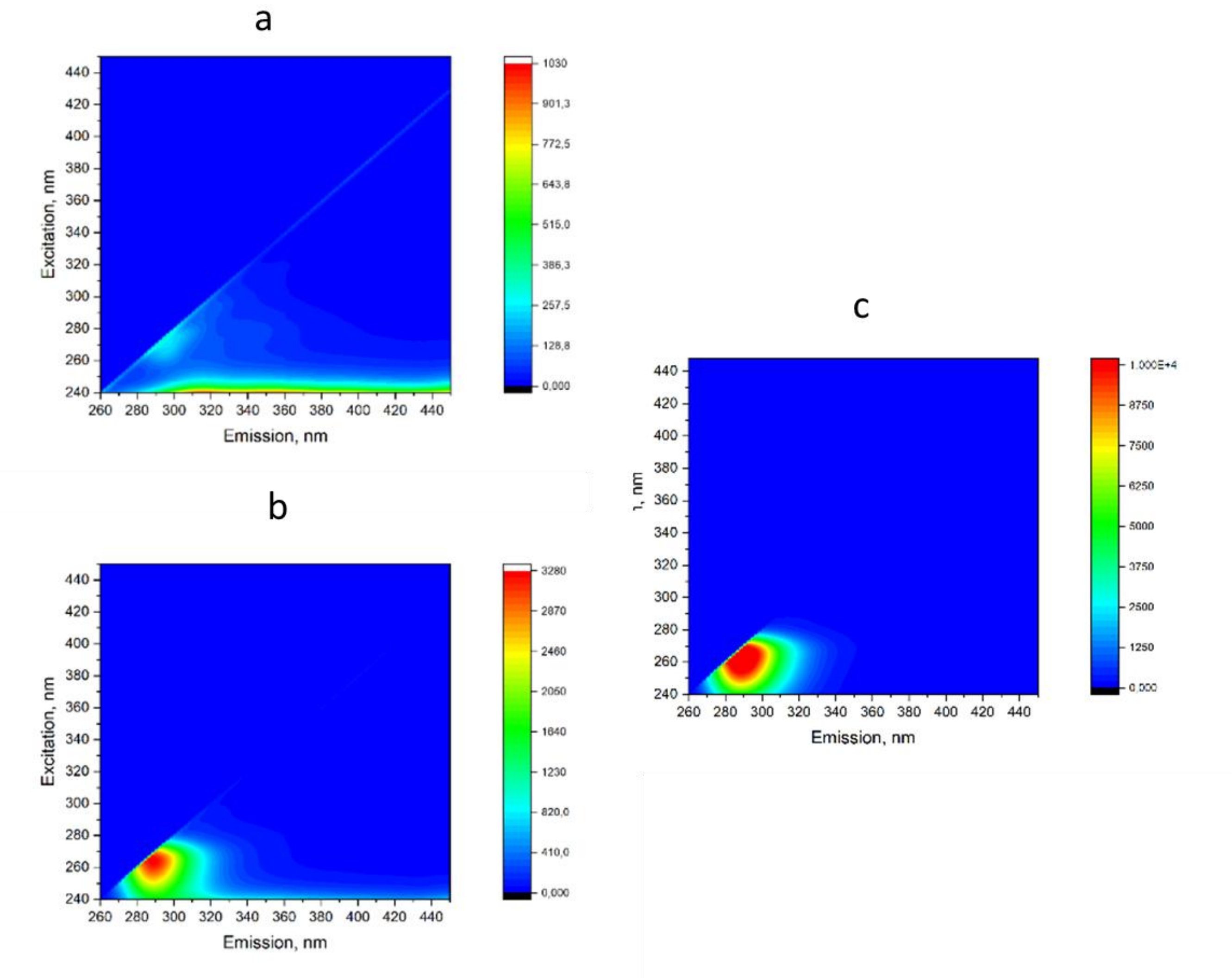

molecules, which have their own fluorescence (Figure 2c).

Supplementary Figure S2. Fluorescence maps of SeNPs (a), SeSo nanocomplexes (b) and sorafenib (So) (c)

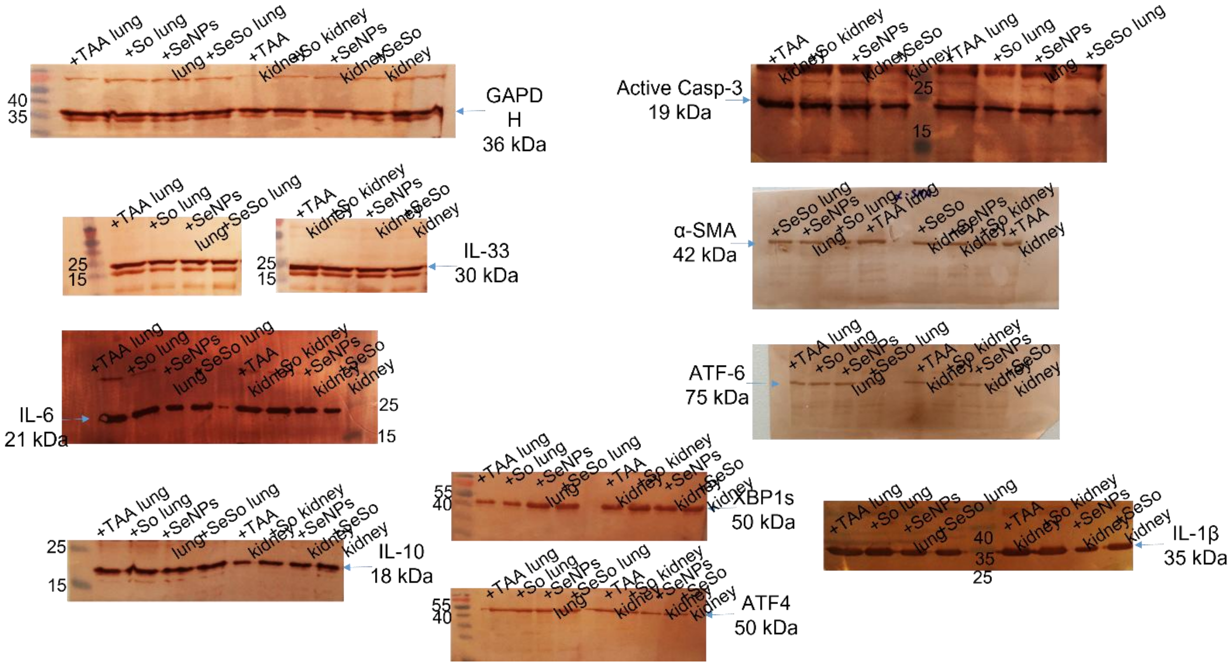

Supplementary Figure S3. The original Western blots.
